# Automated differential diagnosis of dementia syndromes using FDG PET and machine learning

**DOI:** 10.3389/fnagi.2022.1005731

**Published:** 2022-11-02

**Authors:** Matej Perovnik, An Vo, Nha Nguyen, Jan Jamšek, Tomaž Rus, Chris C. Tang, Maja Trošt, David Eidelberg

**Affiliations:** ^1^Department of Neurology, University Medical Center Ljubljana, Ljubljana, Slovenia; ^2^Faculty of Medicine, University of Ljubljana, Ljubljana, Slovenia; ^3^Center for Neurosciences, The Feinstein Institutes for Medical Research, New York, NY, United States; ^4^Department of Genetics, Albert Einstein College of Medicine, New York, NY, United States; ^5^Department of Nuclear Medicine, University Medical Center Ljubljana, Ljubljana, Slovenia

**Keywords:** dementia, differential diagnosis, visual reading, machine learning, FDG PET

## Abstract

**Background:**

Metabolic brain imaging with 2-[^18^F]fluoro-2-deoxy-D-glucose positron emission tomography (FDG PET) is a supportive diagnostic and differential diagnostic tool for neurodegenerative dementias. In the clinic, scans are usually visually interpreted. However, computer-aided approaches can improve diagnostic accuracy. We aimed to build two machine learning classifiers, based on two sets of FDG PET-derived features, for differential diagnosis of common dementia syndromes.

**Methods:**

We analyzed FDG PET scans from three dementia cohorts [63 dementia due to Alzheimer’s disease (AD), 79 dementia with Lewy bodies (DLB) and 23 frontotemporal dementia (FTD)], and 41 normal controls (NCs). Patients’ clinical diagnosis at follow-up (25 ± 20 months after scanning) or cerebrospinal fluid biomarkers for Alzheimer’s disease was considered a gold standard. FDG PET scans were first visually evaluated. Scans were pre-processed, and two sets of features extracted: (1) the expressions of previously identified metabolic brain patterns, and (2) the mean uptake value in 95 regions of interest (ROIs). Two multi-class support vector machine (SVM) classifiers were tested and their diagnostic performance assessed and compared to visual reading. Class-specific regional feature importance was assessed with Shapley Additive Explanations.

**Results:**

Pattern- and ROI-based classifier achieved higher overall accuracy than expert readers (78% and 80% respectively, vs. 71%). Both SVM classifiers performed similarly to one another and to expert readers in AD (F1 = 0.74, 0.78, and 0.78) and DLB (F1 = 0.81, 0.81, and 0.78). SVM classifiers outperformed expert readers in FTD (F1 = 0.87, 0.83, and 0.63), but not in NC (F1 = 0.71, 0.75, and 0.92). Visualization of the SVM model showed bilateral temporal cortices and cerebellum to be the most important features for AD; occipital cortices, hippocampi and parahippocampi, amygdala, and middle temporal lobes for DLB; bilateral frontal cortices, middle and anterior cingulum for FTD; and bilateral angular gyri, pons, and vermis for NC.

**Conclusion:**

Multi-class SVM classifiers based on the expression of characteristic metabolic brain patterns or ROI glucose uptake, performed better than experts in the differential diagnosis of common dementias using FDG PET scans. Experts performed better in the recognition of normal scans and a combined approach may yield optimal results in the clinical setting.

## Introduction

Neurodegenerative dementias are a group of chronic, progressive, and incurable diseases that affect cognition and are caused by the accumulation of abnormally folded proteins, resulting in subsequent neuronal dysfunction and death (Golde et al., 2013). In 2016 there were approximately 45 million people living with dementia. As the population ages, this number is expected to rise and further increase the societal burden (Nichols et al., 2019). The common neurodegenerative dementias include: dementia due to Alzheimer’s disease (AD), dementia with Lewy bodies (DLB), and frontotemporal dementia (FTD; Association, 2018). AD is characterized by abnormal accumulation of amyloid β (Aβ) and hyperphosphorylated tau protein (p-Tau), the typical development of predominantly episodic memory-related symptoms, followed by impairment in other cognitive domains (Lane et al., 2018). DLB is characterized by abnormal accumulation of α-synuclein and a clinical constellation of dementia accompanied by one or more core features of the disease (i.e., parkinsonism, visual hallucinations, fluctuating cognition, and rapid eye movement sleep behavior disorder; Arnaoutoglou et al., 2019). FTD has various underlying causes and presents with three main clinical variants: behavior-variant FTD, non-fluent aphasia, and semantic aphasia. Behavioral variant of FTD is the most common and is characterized by changes in personality and behavior (Bang et al., 2015). Despite differences in main pathology and symptoms among the three most common neurodegenerative dementias, there can be a substantial overlap in concomitant pathology and clinical presentation, especially early in the disease course. Therefore, misdiagnosis is not uncommon even at specialized dementia clinics (Hansson, 2021). Further development and refinement of current biomarkers is therefore required.

Metabolic brain imaging with 2-[^18^F]fluoro-2-deoxy-D-glucose positron emission tomography (FDG PET) is widely accessible, relatively affordable, and a non-invasive imaging technique that provides *in vivo* information about synaptic activity (Stoessl, 2017). It is considered a supportive biomarker for diagnosis of AD, DLB, and FTD (Rascovsky et al., 2011; McKeith et al., 2017; Jack et al., 2018) and for differential diagnosis among them (Nestor et al., 2018). FDG PET scans are usually visually assessed in the clinical setting by an expert reader (either nuclear medicine or neurology specialist, or both). Current guidelines suggest using semi-quantitative techniques based on mass univariate testing to improve diagnostic utility (Varrone et al., 2009). Visual assessments are still prone to errors and inter-rater variability (Ng et al., 2007; Tolboom et al., 2010; Yamane et al., 2014), and therefore, fully automated tools for assessment of FDG PET scans are in development. However, before these tools are integrated into clinic, a head-to-head comparison with expert’s reading is warranted (Nobili et al., 2018).

Different approaches can be used for computer-aided differential diagnosis. Multivariate analysis approaches, such as scaled subprofile model/principal component analysis (SSM/PCA) applied to FDG PET images, can reveal characteristic metabolic brain patterns, which expressions can be prospectively quantified on a single case basis (Spetsieris and Eidelberg, 2011). AD-related pattern (ADRP; Perovnik et al., 2022a), DLB-related pattern (DLBRP; Perovnik et al., 2022b) and FTD-related pattern (FTDRP; Rus et al., 2021) have been identified and validated in different clinical cohorts in the past. Similarly, a pattern of default mode network (DMN)—a dominant resting-state network in healthy individuals, which is affected also in the pathogenesis of neurodegenerative dementias—has been characterized with FDG PET (Spetsieris et al., 2015). Based on the expression of metabolic brain patterns, we can very accurately distinguish between patients with dementia and normal controls (NCs; Perovnik et al., 2022a,b). However, it was also observed that the information of a single pattern’s expression score may not be sufficient to distinguish between different neurodegenerative dementias accurately (Katako et al., 2018). While the advantage of using the information on multiple metabolic brain patterns’ expression in conjunction with a simple machine learning algorithm (i.e., logistic model regression) has been shown in neurodegenerative parkinsonisms (Tang et al., 2010; Tripathi et al., 2016; Rus et al., 2020; Papathoma et al., 2022), their utility remains to be investigated for the differential diagnosis of neurodegenerative dementias.

In recent years, various machine learning algorithms, such as support vector machine (SVM) or deep neural networks have been developed and refined to aid in brain scan classification: where the features (e.g., regions) necessary to make a correct prediction are learned from the data (Myszczynska et al., 2020). However, many such studies focused only on Alzheimer’s disease, distinguishing between dementia and controls or mild cognitive impairment and controls (Borchert et al., 2021). In the clinical setting, the diagnostic dilemma is usually not limited to distinguishing between AD and healthy controls, but among the various neurodegenerative dementias. Before computer-aided systems can be successfully applied to the clinical setting, they need to be adjusted to recognize different neurodegenerative dementia syndromes (Burgos and Colliot, 2020).

In this study, we aimed to build two multi-class machine learning classifiers based on two different sets of features for the differentiation of FDG PET scans of patients with the most common dementia syndromes (AD, DLB, and FTD) and NC, and to compare the algorithms’ classification accuracy to gold standard diagnosis and expert visual reading. We also explored the characteristic features of each disease type.

## Materials and methods

### Study design

**Figure 1:**
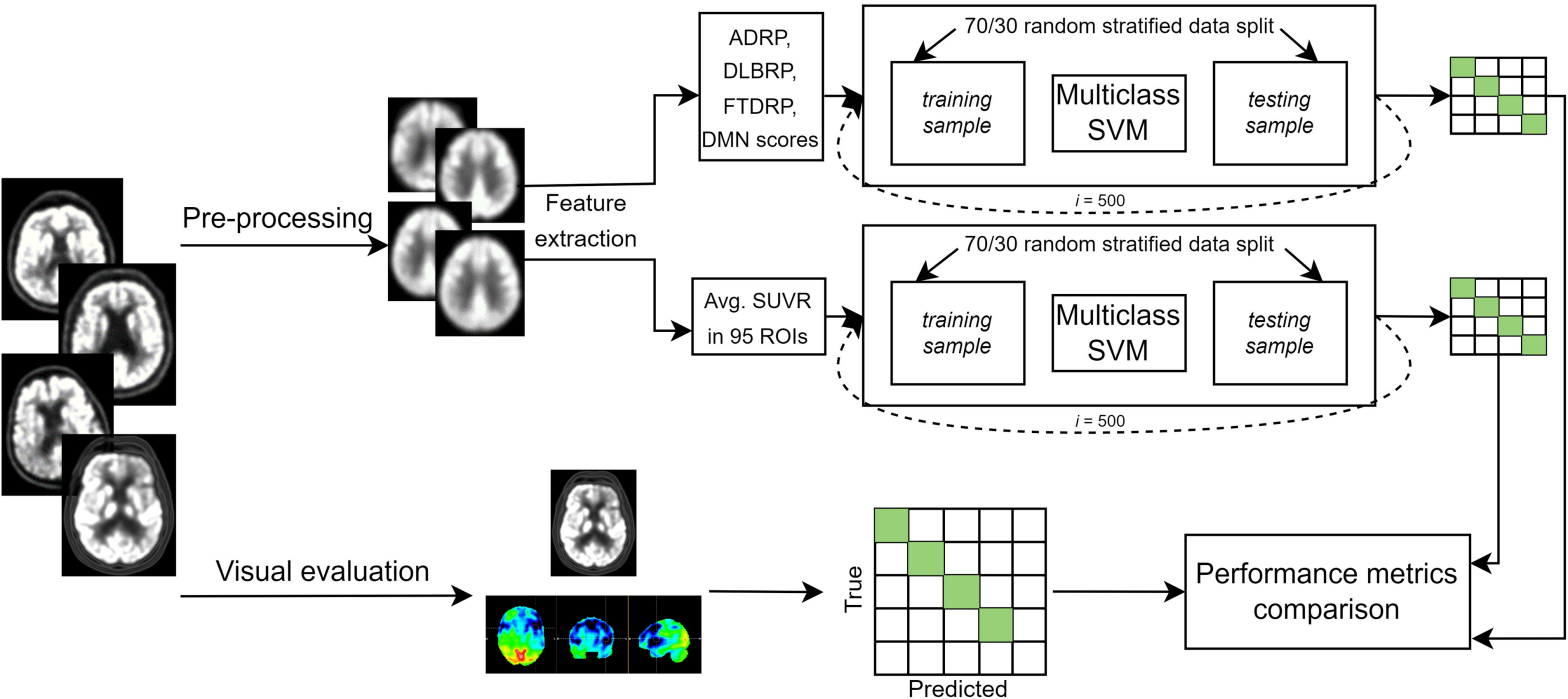
Flowchart of the general workflow. Top: 2-[^18^F]fluoro-2-deoxy-D-glucose positron emission tomography (FDG PET) scans from three dementia cohorts and healthy participants underwent pre-processing, and two set of features [pattern expression scores and standard uptake value ratio (SUVR) in 95 regions of interest (ROIs)] were extracted. Two multi-class support vector machine (SVM) models were built to classify scans either as dementia due to Alzheimer’s disease (AD), dementia with Lewy bodies (DLB), frontotemporal dementia (FTD) or normal. Most common label after 500 iterations was compared to gold standard, and performance metrics calculated. Bottom: FDG PET scans were visually evaluated by two expert readers. Scans could be read either as AD, DLB, FTD, normal or other (inconclusive or other neurodegenerative pattern). Labels were compared to gold standard and performance metrics were calculated. Clinical diagnosis at follow-up or cerebrospinal fluid (CSF) biomarker for Alzheimer’s disease was considered a gold standard. ADRP, Alzheimer’s disease-related pattern; DLBRP, dementia with Lewy bodies-related pattern; FTDRP, frontotemporal dementia-related pattern; DMN, default mode network pattern.

In this study we analyzed 206 FDG PET scans from three dementia cohorts and healthy participants. Diagnosis at follow-up (M = 25 months, SD = 20 months) or cerebrospinal fluid (CSF) biomarkers for Alzheimer’s disease was considered a gold standard. FDG PET scans were first visually evaluated by expert readers (nuclear medicine and neurology specialist) and diagnostic performance was assessed. Then, we built two multi-class machine learning classifiers – one based on pattern expression values and the other on regions of interest (ROIs) uptake values – and assessed their diagnostic performance. The performance of the classifiers was compared to the one achieved by visual reading. Lastly, we examined the most important features of the ROI-based classifier by identifying the most important regional features on the entire dataset and by visualizing class-specific regional importance. Study workflow is schematically illustrated in Figure 1.

### Participants

We analyzed three dementia cohorts (63 AD, 79 DLB, and 23 FTD) and 41 normal controls (NCs). All participants underwent FDG PET brain imaging between January 2010 and April 2019 at the University Medical Center Ljubljana (UMCL), Slovenia. Patients with AD fulfilled diagnostic criteria for probable AD dementia (amnestic presentation; McKhann et al., 2011) and had the diagnosis confirmed by cerebrospinal fluid (CSF) analysis of Aβ42 or Aβ42/Aβ40, p-tau, and t-tau according to the recent National Institute of Aging-Alzheimer’s Association research framework (Jack et al., 2018). A detailed CSF procedure has been described previously (Perovnik et al., 2022a). Patients with DLB were diagnosed according to the 4th Consensus report of the DLB Consortium (McKeith et al., 2017), followed by a diagnosis confirmation at an office visit conducted at least 12 months after the onset of symptoms. Patients with FTD were diagnosed with possible FTD according to the International Behavioral Variant FTD Consortium criteria (Rascovsky et al., 2011) and had their diagnosis confirmed at a follow-up visit conducted at least 12 months after the onset of symptoms. AD and DLB cohorts and their FDG PET scans included in this study have appeared previously (Perovnik et al., 2022a,b). NCs completed clinical, neuropsychological, and FDG PET brain imaging for purposes of an earlier research project (Tomše et al., 2017).

### Image acquisition

FDG PET images were acquired at the Department of Nuclear Medicine, UMCL, with Siemens Biograph mCT PET/CT (Siemens Healthineers, Erlangen, Germany) according to European Association of Nuclear Medicine guidelines (Varrone et al., 2009). The detailed procedure has been described previously (Tomše et al., 2017).

### Visual reading

All FDG PET images were assessed visually by a neurology and nuclear medicine specialist working in tandem. First, reconstructed images were visually evaluated, and then the physicians assessed the scans with semi-quantitative analysis using Scenium©, a Siemens software that is a part of Syngo.via Neurology package (Siemens CTI Molecular Imaging, Knoxville, TN, United States). The diagnosis was made by recognizing the typical topography of metabolic brain changes caused by neurodegenerative syndromes (Brown et al., 2014). The scans could be read either as one of the three included dementia subtypes (AD, DLB, FTD), normal, inconclusive, or exhibiting a pattern of another neurodegenerative disease.

### Image pre-processing

FDG PET scans were pre-processed with SPM12 (Wellcome Trust Centre for Neuroimaging, Institute of Neurology, London, United Kingdom) running on Matlab R2019a (Mathworks Inc., Natick, MA, United States) using an in-house pipeline. We performed brain extraction by segmenting the skull based on a tissue probability map. Then we spatially normalized scans onto a PET template in the Montreal Neurological Institute brain space. Finally, the images were smoothed with an isotropic 3D Gaussian kernel of 10 mm FWHM.

### Feature extraction

We calculated the expression of previously identified and validated specific metabolic patterns related to AD (Perovnik et al., 2022a), DLB (Perovnik et al., 2022b), and FTD (Rus et al., 2021). We also calculated expression levels for the normal metabolic DMN expression as described previously (Spetsieris et al., 2015). The scores were obtained by calculating the dot product between logarithmically transformed and double centered patient’s scan and pattern vector using the topographic profile rating procedure described elsewhere (Eidelberg, 2009). The scores were Z-transformed based on the mean pattern expression, and standard deviation of subjects’ scores in the NC identification cohort were used to identify each pattern.

We extracted mean glucose metabolism scaled on the global uptake as the second set of features. It was shown previously that global normalization outperforms cerebellar normalization in the differential diagnosis of different types of dementia (Dukart et al., 2010). We used two different Automated Anatomic Labeling (AAL) atlases for value extraction. First, we used 95 regions of interest (ROIs; Tzourio-Mazoyer et al., 2002) with modifications as described previously (Ko et al., 2018). A complete list of names and abbreviations is provided in Spetsieris and Eidelberg (2021). Second, we used 166 ROIs as provided previously (Rolls et al., 2020) with additional custom-made pons region and the mean uptake for anterior cingulate cortices and thalami were additionally calculated for a total of 171 ROIs.

### Classification model

SVM analysis for multi-class classification with linear kernel was implemented in Matlab R2019a using *fitcecoc* function, and two different sets of features were used (pattern expression values and ROI glucose uptake). In each of the 500 iterations, the data were randomly split between training and testing sets (70,30) in a stratified fashion, retaining the original group balance. Each model was applied prospectively to the testing data in that iteration and the labels for each case in the testing were obtained. The final label was defined as the most common label across 500 iterations assigned to a scan. Because some of the participants (20 AD, 20 DLB, 10 FTD, and 29 NC) were also used to identify respective patterns, these scans were only used in training sets in the pattern-based SVM model to avoid data leakage. For the sake of accurate comparison between the SVM models, we repeated the analyses for the ROI-based SVM model using just the reduced dataset.

### Model explanations

We assessed feature importance using neighborhood component analysis (NCA) for classification. Initially, we used the entire dataset and Limited memory Broyden–Fletcher–Goldfarb–Shanno (LBFGS) algorithm (Liu and Nocedal, 1989) to obtain the feature weights. The latter correspond to values that minimize an objective function measuring the average leave-one-out classification loss over the data. We explored the effect of the number of features included in the model on the final classification accuracy for each group by adding features to the model from most to least important. Then, we also explored the training set’s effect on each run’s feature selection procedure by identifying the best non-zero lambda value corresponding to the minimum average loss and plotting a frequency histogram. Lambda value was optimized in each iteration.

Furthermore, we employed Shapley Additive Explanations (SHAP; Lundberg and Lee, 2017) to estimate individual-level explanations of ROI-based models using shapley function with an extension of the kernel SHAP algorithm to address feature dependency in our dataset (Aas et al., 2021). SHAP is a game-theoretical concept used to assess the contribution of each feature to the final model decision in a particular case (Lundberg and Lee, 2017). We calculated Shapley values in the training set for each run and plotted group absolute mean values on the AAL template to visualize the class-specific regional importance.

Feature selection was done as a complementary exploration of the ROI-based SVM model and was not included in the final pipeline for which performance results are presented.

### Statistical analysis

One-way analysis of variance (ANOVA) with *post-hoc* Bonferroni corrected *t*-test was used to examine the differences in age, MMSE, and disease duration between groups. Fisher’s exact test for count data was used to examine differences in sex distribution between groups. Results were considered significant at *p* < 0.05 (two-tailed). Statistical analyses were conducted in RStudio version 1.3.1093 and R version 3.6.0 (R Core Team, 2019). We also calculated overall accuracy, true positive (TP), false positive (FP), false negative (FN), and true negative (TN) cases based on the confusion matrix. Furthermore, we calculated specificity (TN/(TN + FP)), precision (TP/(TP + FP)), and sensitivity (TP/(TP + FN)); due to class imbalance the F1 score ((2*(Precision*Sensitivity))/(Precision+Sensitivity)) was also calculated for each class. Receiver operating characteristic (ROC) curves were plotted for each class separately in one vs. all design, and the areas under the ROC curves (AUCs) were calculated.

## Results

The subject’s demographics and clinical data are presented in Table 1, and the general workflow is presented in Figure 1. There was a significant age difference between groups [*F*(3, 202) = 18.45, *p* < 0.001]; *post-hoc* tests indicated that AD and DLB were significantly older from FTD (both *p* < 0.01) and NC (both *p* < 0.001). Age did not differ between AD and DLB (*p* = 0.50) or between FTD and NC (*p* = 1.00). Sex distribution differed between DLB and NC groups (*p* = 0.006) but not between any of the other pairwise comparisons (all *p* > 0.42). Disease duration did not differ significantly between the three dementia groups [*F*(2, 145) = 1.62, *p* = 0.20]. There was a significant difference in MMSE scores between groups [*F*(3, 160) = 39.3, *p* < 0.001] and *post-hoc* tests indicated that DLB had higher scores than AD (*p* = 0.002), but there was no significant difference between DLB and FTD (*p* = 1.00) or AD and FTD (*p* = 0.055). NC had higher MMSE scores than the three dementia groups (all *p* < 0.001).

**Table 1:** Demographic and clinical data.

	AD	DLB	FTD	NC
*N*	63	79	23	41
Age (y)	72.9 (8.8)	75.2 (6.5)	66.5 (9.9)	65.3 (7.0)
Sex (f/m)	31/32	28/51	13/10	28/13
Disease duration (y)	3.6 (2.3) (*n* = 54)	3.9 (2.1) (*n* = 74)	3.0 (2.0) (*n* = 20)	/
MMSE	18.0 (5.1) (*n* = 59)	21.1 (5.0) (*n* = 54)	21.1 (5.5) (*n* = 20)	29.0 (1.0) (*n* = 32)

All the data are presented as mean (SD). AD, dementia due to Alzheimer’s disease; DLB, dementia with Lewy bodies; FTD, frontotemporal dementia; NC, normal control; MMSE, Mini-Mental State Examination.

### Visual reading

**Figure 2:**
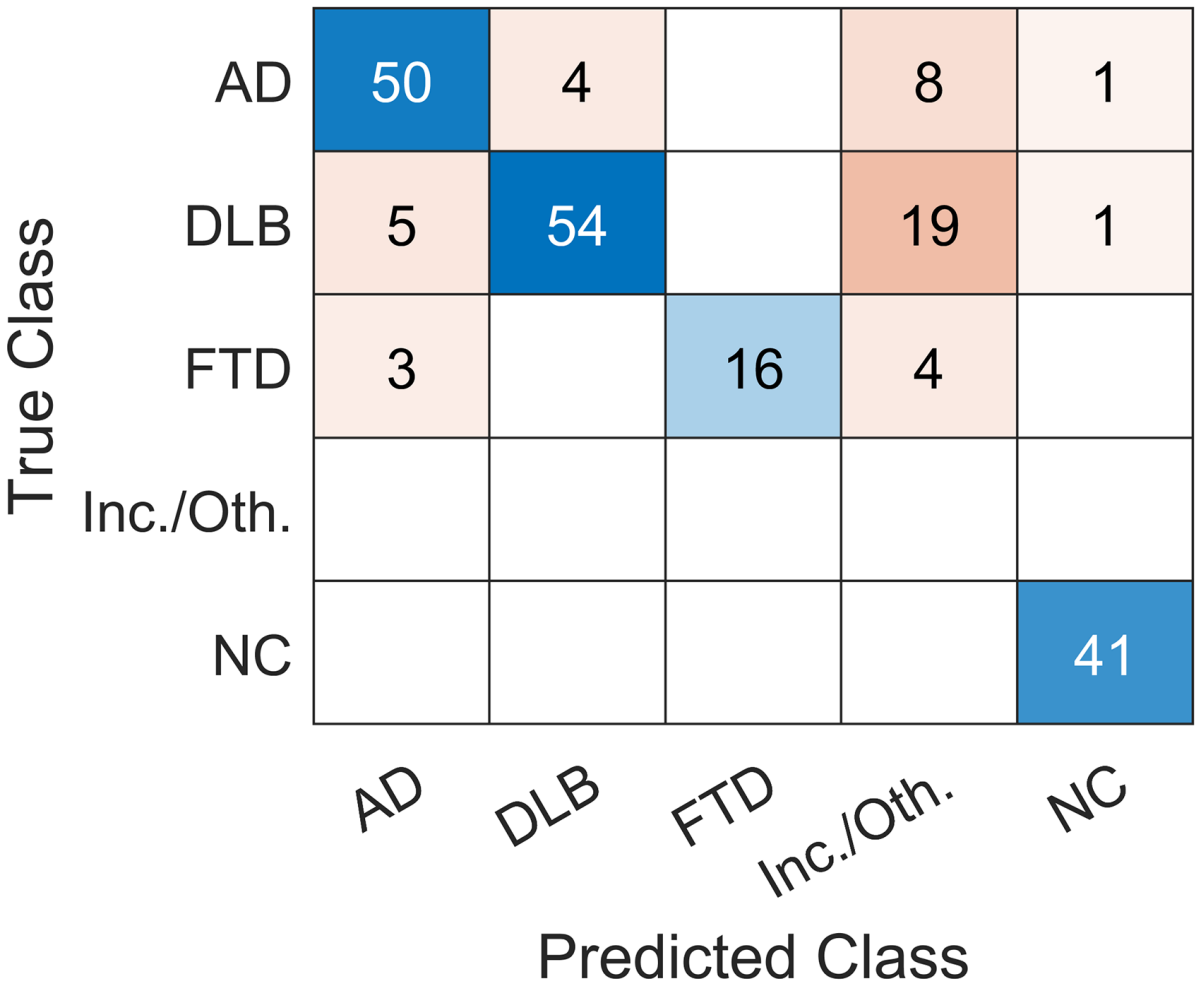
Confusion matrix of the classification results using visual reading. Expert readers correctly diagnosed 161 out of 206 cases and achieved 78% overall accuracy. Scans were read (Predicted Class) either as dementia due to Alzheimer’s disease (AD), dementia with Lewy bodies (DLB), frontotemporal dementia (FTD), normal (NC) or other (inconclusive (inc.) or other (oth.) neurodegenerative pattern). The label was compared to gold standard (True Class).

Expert readers correctly classified 161 out of 206 cases and achieved 78% overall accuracy (Table 2A; Figure 2). They had high specificity and precision but limited sensitivity in the three dementia groups: AD (specificity 94%, precision 86%, sensitivity 79%), DLB (specificity 97%, precision 93%, sensitivity 68%), and FTD (specificity 100%, precision 100%, sensitivity 70%). Expert readers achieved high specificity, precision, and sensitivity in the NC group (specificity 99%, precision 95%, sensitivity 100%).

**Table 2:** Performance metrics for visual reading and the three machine learning classifiers.

A	Entire dataset				
	AD				DLB				FTD				NC				Overall acc.
***N***	63				79				23				41				
	F1	Sp	Pr	Se	F1	Sp	Pr	Se	F1	Sp	Pr	Se	F1	Sp	Pr	Se	
Visual read	0.83	94	86	79	0.79	97	93	68	0.82	100	100	70	0.98	99	95	100	78
Pattern-based classifier	–	–	–	–	–	–	–	–	–	–	–	–	–	–	–	–	–
95 ROIs-based classifier	0.83	92	82	84	0.86	94	89	82	0.91	99	95	87	0.89	95	83	95	86
171 ROIs-based classifier	0.81	92	81	82	0.86	93	88	85	0.91	99	95	87	0.89	96	84	93	86
**B**	**Reduced dataset**																
	**AD**				**DLB**				**FTD**				**NC**				**Overall acc.**
***N***	**43**				**59**				**13**				**12**				
	**F1**	**Sp**	**Pr**	**Se**	**F1**	**Sp**	**Pr**	**Se**	**F1**	**Sp**	**Pr**	**Se**	**F1**	**Sp**	**Pr**	**Se**	
Visual read	0.78	92	82	74	0.78	94	91	68	0.63	100	100	46	0.92	98	86	100	71
Pattern-based classifier	0.74	87	74	74	0.81	87	84	78	0.87	100	100	77	0.71	93	58	82	78
95 ROIs-based classifier	0.78	88	77	79	0.81	90	86	77	0.83	99	91	77	0.75	93	60	100	80
171 ROIs-based classifier	0.78	89	79	81	0.84	91	89	80	0.92	99	92	92	0.77	84	63	100	83

Performance metrics are calculated on (A) the entire dataset and on (B) the reduced dataset, where we excluded patients used for pattern identification from the testing set. Specificity, precision, sensitivity, and overall accuracy are presented as %. AD, dementia due to Alzheimer’s disease; DLB, dementia with Lewy bodies; FTD, frontotemporal dementia; NC, normal control; ROI, a region of interest; Sp, specificity; Pr, precision; Se, sensitivity; Acc, accuracy.

### Pattern-based classifier

**Figure 3:**
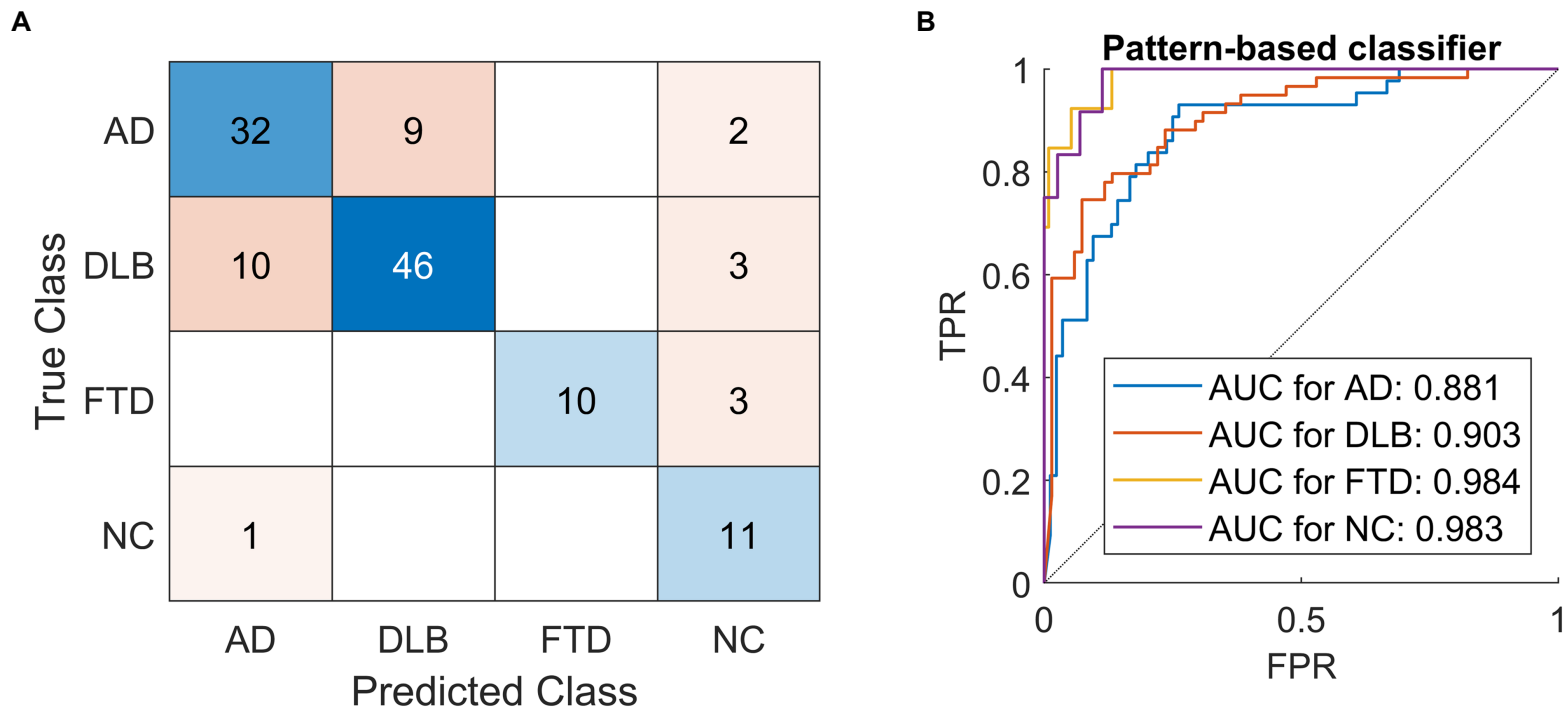
The classification results using a pattern-based support vector machine classifier. A classifier based on pattern expression values and multi-class support vector machine (SVM) correctly diagnosed 99 out of 127 cases and achieved 78% overall accuracy. **(A)** Confusion matrix of labels predicted by the SVM (Predicted Class) compared to gold standard (True Class). **(B)** One vs. all receiver operating characteristic (ROC) curves for the four possible labels. AD, dementia due to Alzheimer’s disease; DLB, dementia with Lewy bodies; FTD, frontotemporal dementia; NC, normal control; AUC, area under the curve; TPR, true positive rate; FPR, false positive rate.

A classifier based on pattern expression values and multi-class SVM correctly classified 99 out of 127 cases and achieved 78% overall accuracy (Table 2B; Figure 3A). Note that 79 cases (20 AD, 20 DLB, 10 FTD, and 29 NC) were not included in the testing set to avoid data leakage, as those scans were used to derive the patterns. Pattern-based SVM had high specificity and precision but lower sensitivity in DLB (specificity 87%, precision 84%, sensitivity 78%, AUC = 0.90) and FTD (specificity 100%, precision 100%, sensitivity 77%, AUC = 0.98) groups. It had high specificity and lower precision and sensitivity in AD (specificity 87%, precision 74%, sensitivity 74%, AUC = 0.88) group, and high specificity and sensitivity with lower precision in NC (specificity 93%, precision 58%, sensitivity 82%, AUC = 0.98) group. ROC curves for pattern-based classifier are plotted for each class separately (Figure 3B).

### Regions of interest-based classifier

**Figure 4:**
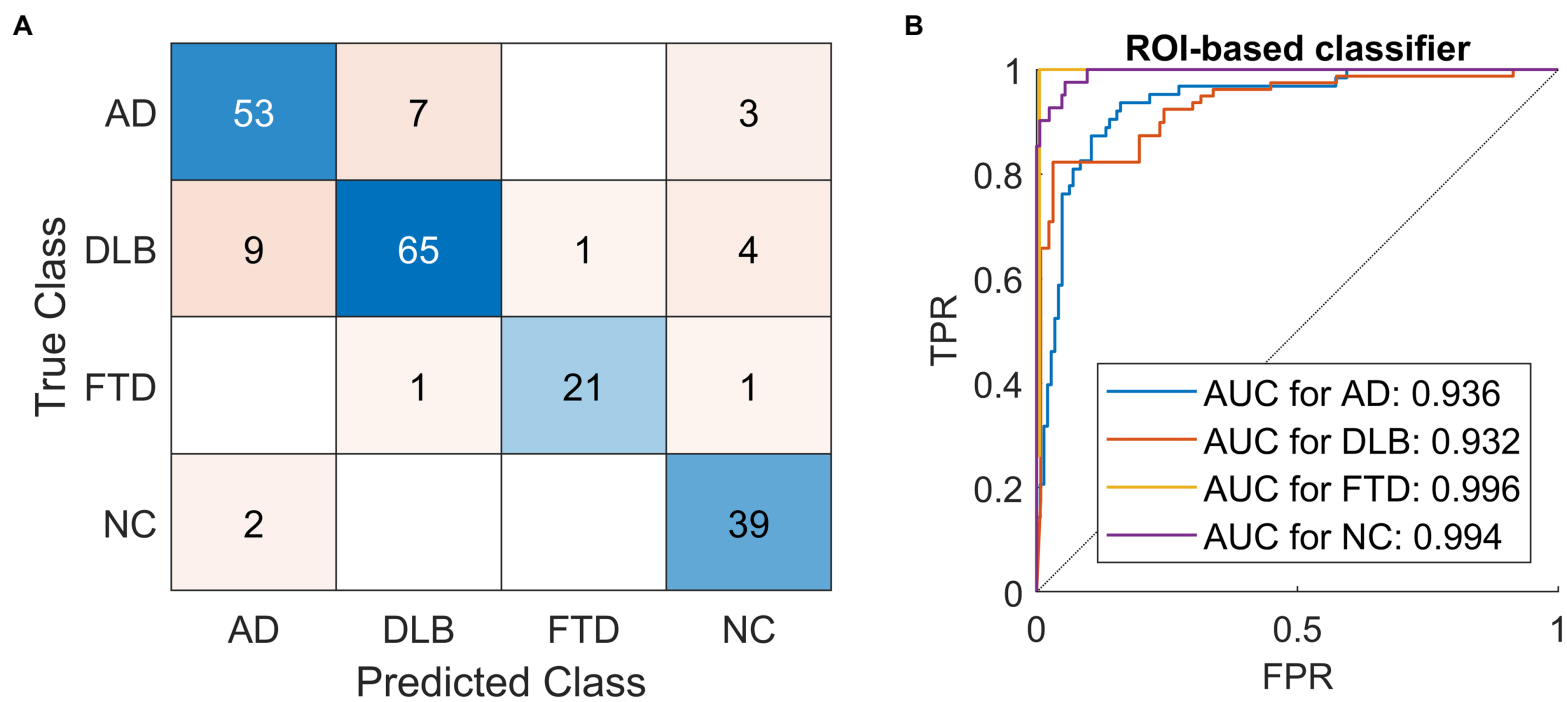
The classification results using a region of interest-based support vector machine classifier. A classifier based on 95 regions of interest (ROIs) and multi-class support vector machine (SVM) correctly diagnosed 177 out of 206 cases and achieved 86% overall accuracy. **(A)** Confusion matrix of labels predicted by the SVM (Predicted Class) compared to gold standard (True Class). **(B)** One vs. all receiver operating characteristic (ROC) curves for the four possible labels. AD, dementia due to Alzheimer’s disease; DLB, dementia with Lewy bodies; FTD, frontotemporal dementia; NC, normal control; ROI, region of interest; AUC, area under the curve; TPR, true positive rate; FPR, false positive rate.

A classifier based on 95 ROIs and multi-class SVM correctly classified 177 out of 206 cases and achieved 86% overall accuracy (Table 2A; Figure 4A), and had high specificity, sensitivity, and precision in all four groups: AD (specificity 92%, precision 82%, sensitivity 84%, AUC = 0.94), DLB (specificity 94%, precision 89%, sensitivity 82%, AUC = 0.93), FTD (specificity 99%, precision 95%, sensitivity 87%, AUC = 1.00), and NC (specificity 95%, precision 83%, sensitivity 95%, AUC = 0.99). ROC curves are plotted for each class separately (Figure 4B). A classifier based on 171 ROIs and multi-class SVM correctly diagnosed 176 out of 206 cases and achieved 86% overall accuracy with similar specificity, sensitivity, and precision values across groups as a classifier based on 95 ROIs.

F1 scores for all four approaches are reported in Table 2. The scores are presented using the entire dataset (Table 2A) and for the sake of accurate comparison between the models also by using the reduced dataset in which 79 scans used for pattern identification were restricted to the training set (Table 2B).

### Model explanations

**Figure 5:**
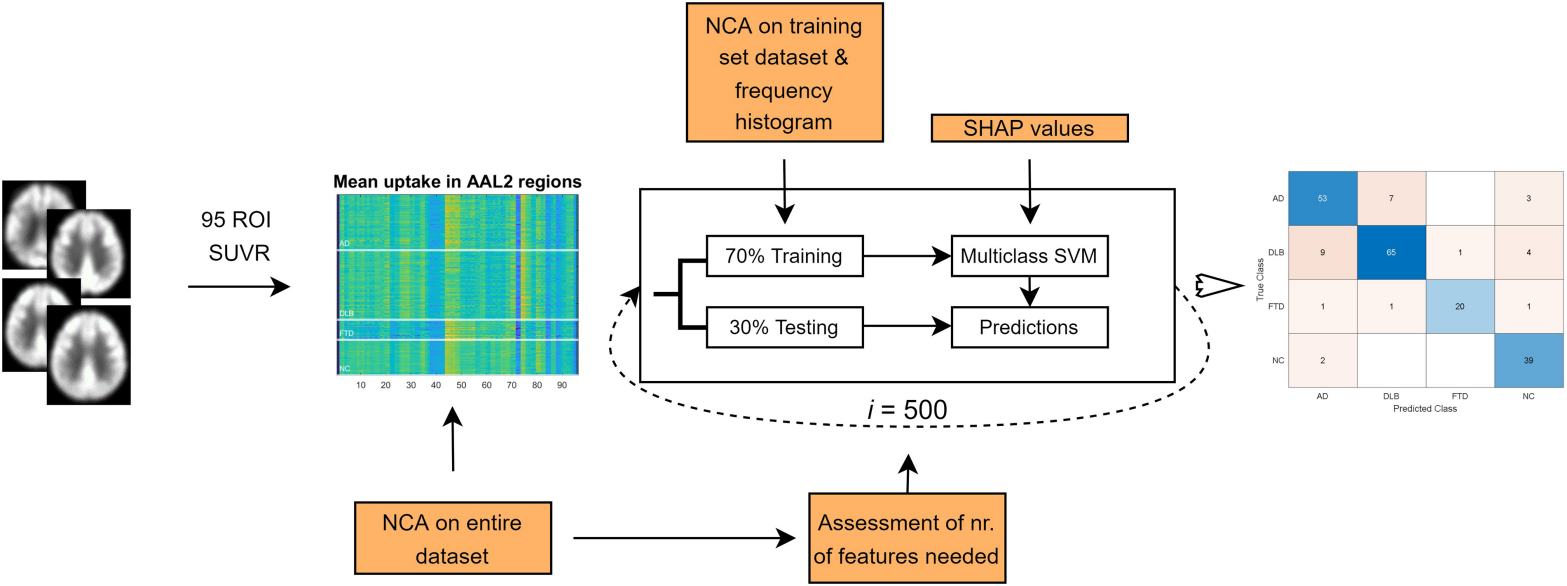
Flowchart depicting three different approaches to the explanation of the model. Initially, we used the entire dataset and assessed feature importance using neighborhood component analysis (NCA). The weights were used to rank the features from most to least important. NCA was then performed separately on just the training set for each iteration, and the retained features were plotted on a frequency histogram. To explain the support vector machine (SVM) model we employed Shapley Additive Explanations (SHAP). ROI, region of interest; SUVR, standard uptake value ratio; AAL, automated anatomic labeling atlas.

We have tried to explain the ROI-based classifiers on three different levels and the schematic is shown in Figure 5.

**Figure 6:**
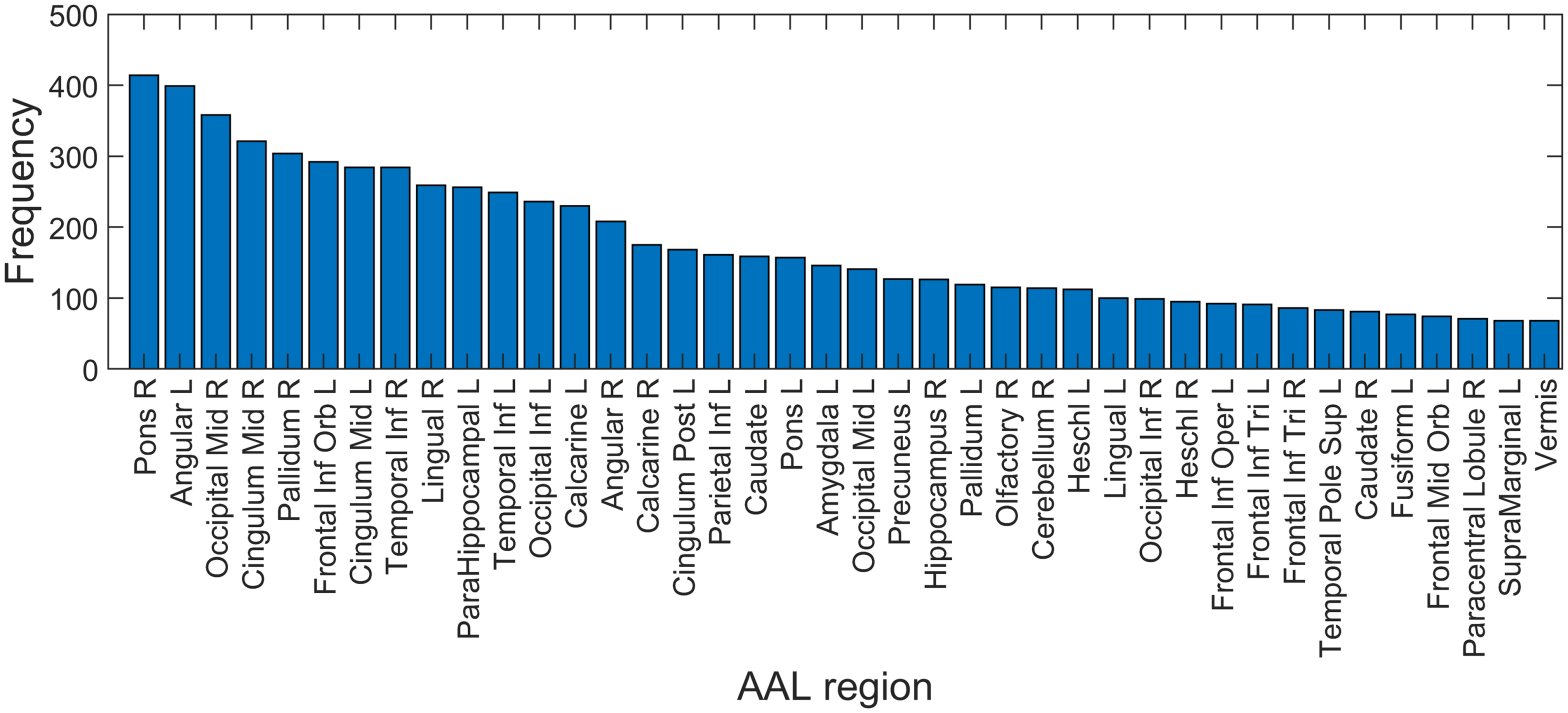
Feature selection frequency histogram of 40 most important features. Feature selection was performed on training set in each iteration using neighborhood component analysis (NCA), identifying the best non-zero lambda value corresponding to the minimum average loss. AAL, automated anatomic labeling; L, left; R, right.

Based on the NCA results on the entire dataset, the regions with feature weights exceeding 0.3 were: bilateral angular gyri, left pallidum, bilateral calcarine sulci, pons, left middle cingulum, bilateral superior frontal cortex, right hippocampus, left middle occipital cortex, left inferior temporal lobe, left inferior occipital cortex and left inferior parietal cortex. Similar regions were seen on the frequency histogram with feature selection on just the training set in each of the 500 iterations (Figure 6).

**Figure 7:**
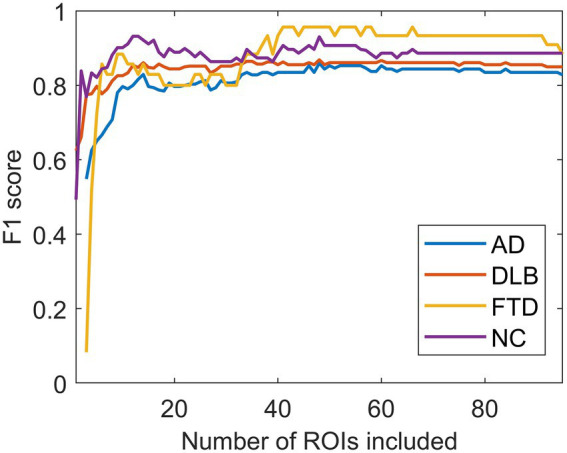
The effect of the number of regions of interest included on the F1 scores. F1 scores reached their maximal values after including the 40 most important regions of interest (ROIs). AD, dementia due to Alzheimer’s disease; DLB, dementia with Lewy bodies; FTD, frontotemporal dementia; NC, normal controls.

F1 scores reached their maximal values after including the 40 most important ROIs based on the NCA results obtained on the entire dataset (Figure 7).

**Figure 8:**
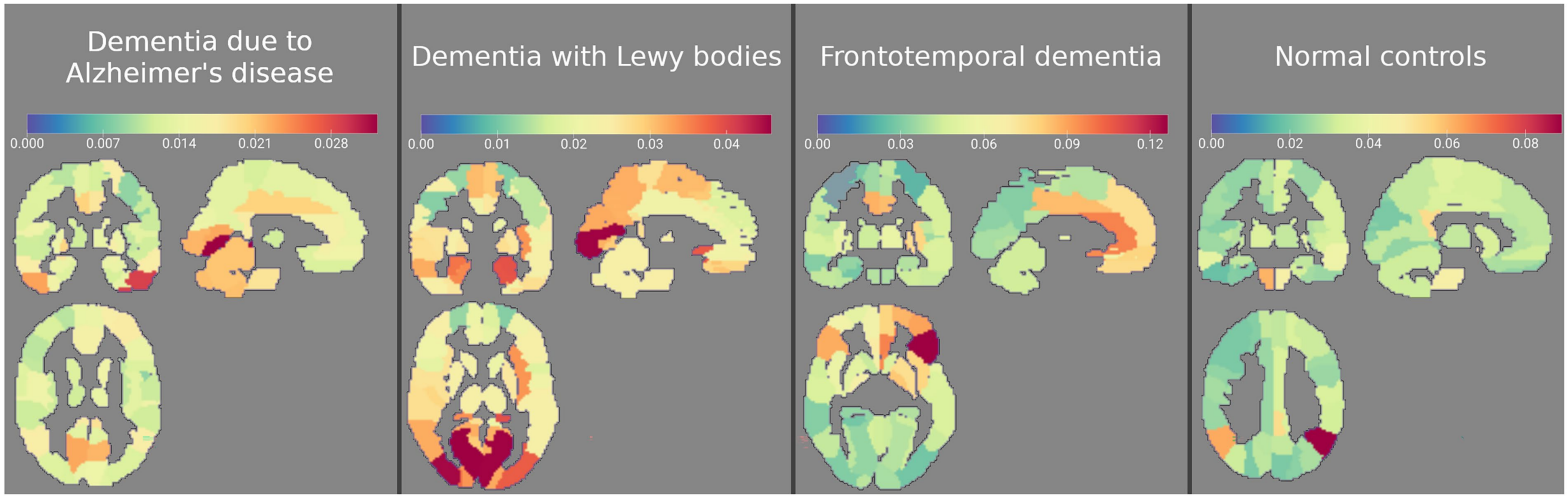
Absolute average Shapley values for four groups plotted on an AAL template. The most important features for dementia due to Alzheimer’s disease (AD) classification included bilateral temporal cortices, cerebellum, bilateral lingual, and calcarine sulci, for dementia with Lewy bodies (DLB) occipital cortices, hippocampi and parahippocampi, amygdala, and middle temporal lobes, for frontotemporal dementia (FTD) bilateral frontal cortical regions, middle cingulum and anterior cingulum, and for normal controls (NC) bilateral angular gyri, pons, and vermis. Regions (features) are color-coded blue to red from least to most important.

Absolute average SHAP values plotted on an AAL template for each of the four groups are shown in Figure 8. The most important features for AD classification included bilateral temporal cortices, cerebellum, bilateral lingual, and calcarine sulci, for DLB classification included occipital cortices, hippocampi and parahippocampi, amygdala, and middle temporal lobes and for FTD classification included bilateral frontal cortical regions, middle cingulum, and anterior cingulum. The most important features for NC classification included bilateral angular gyri, pons, and vermis.

## Discussion

In this study, we applied a multi-class SVM, based either on the expression of disease-specific metabolic brain patterns or ROI glucose uptake values, as features to classify FDG PET brain scans of participants with three different dementia syndromes (AD, DLB, and FTD) and NC. The classification accuracies, F1 scores, specificity and sensitivity between the models were then compared to the experts’ visual reading.

Pattern- and ROI-based classifier achieved higher overall accuracy compared to the expert readers (78 and 80% respectively, vs. 71%). Looking at the entire dataset (not excluding the patients used for pattern identification from the testing set), the ROI-based SVM model also achieved higher overall accuracy than visual reading (86% vs. 78%, respectively). Both SVM classifiers performed similarly to one another and to expert readers in AD (F1 = 0.74, 0.78, and 0.78) and DLB (F1 = 0.81, 0.81, and 0.78) groups. However, in DLB group expert readers achieved lower sensitivity (68%) than SVM classifiers (77%–85%). SVM classifiers consistently outperformed expert readers in FTD group (F1 = 0.87, 0.83, and 0.63), mostly due to the lower sensitivity of expert readers (46% in reduced and 70% in entire dataset vs. 77%–92%). Lower sensitivity of visual assessment has been reported previously (Rabinovici et al., 2011; Tripathi et al., 2014; Etminani et al., 2021). Expert readers, however, achieved higher specificity and precision in AD, DLB, and FTD groups compared to the two SVM classifiers. Lower sensitivity in combination with high specificity shows that expert readers needed to recognize clear visual features of the disease to make a diagnosis and when they made a final call, they had less false positive readings than SVM models. In the NC group, SVM classifiers performed worse than expert readers (F1 = 0.71, 0.75, and 0.92). Expert readers were less likely to classify the scan belonging to a diseased individual as normal. Indeed, only two scans belonging to diseased individuals were read as normal. However, expert readers could classify the scans that showed some pathological changes as inconclusive, while SVM models were “forced” to make a diagnostic decision. On one hand, that resulted in more diseased scans being misclassified as normal but on the other, it increased the number of scans that were correctly classified. Taken together, SVM classifiers outperformed visual readers in overall accuracy, but our results show that a combination of both approaches offers complementary information. In borderline cases, a decision from the SVM classifier might help the physician to make a final diagnosis and a combination of expert knowledge and computer-aided assessment might be superior to either approach alone.

While the ROI-based classifier needed 40 features to reach the maximal classification accuracy, we have also shown that similar performance to visual reading can be made using only the expression of four characteristic metabolic brain patterns. In addition, previous studies have shown that pattern expression scores correlate with measurements of cognitive impairment in AD and DLB (Perovnik et al., 2022a,b), and higher expression values are predictive of conversion from MCI to AD (Blazhenets et al., 2019). In contrast to the ROI-based approach, pattern expression values can be more easily interpreted by a physician who would want to use the multi-class SVM as a supportive tool to aid the differential diagnosis.

Several studies have explored the utility of FDG PET for dementia differential diagnosis using computer-aided approaches. Xia et al. (2014) reported an overall accuracy of 95% with high sensitivity and specificity for the classification of AD, FTD, and NC using FDG PET scans. Díaz-Álvarez et al. (2022) utilized genetic algorithms [which are population-based dimensionality reduction techniques that aim to maximize the classification performance while keeping the number of features low (Deb et al., 2002)], for feature selection with K-Nearest Neighbor, and the naïve Bayes model for classification of AD, FTD, primary progressive aphasia variants, and NC, by using FDG PET scans. This approach achieved 86–93% classification accuracies in one vs. one design. Etminani et al. (2021), on the other hand, employed a deep learning model and included AD, DLB, MCI-AD, and NC scans in their study and reported high AUC values for AD (0.96), DLB (0.96), and NC (0.95). However, algorithms that include information on all the most common dementias are needed for successful implementation in the clinical workup. Mattila et al. (2012) developed a disease state index (DSI) classifier for five-class classification [AD, DLB, FTD, Vascular dementia (VaD) and subjective memory complaint (SMC)]. DSI is a data-driven model for dementia differential diagnosis utilizing a combination of clinical tests, CSF biomarkers, and MRI features to aid clinicians, which was shown to have an accuracy of 75% (Tong et al., 2017). FDG PET imaging can increase classification accuracy even further (Gjerum et al., 2020). Indeed, using only FDG PET data with a DSI classifier, high AUC values for AD (0.84–0.87), DLB (0.84–0.97), and FTD (0.87–0.97) were reported. The AUC values were lower for the VaD group, where MRI outperformed FDG PET (Gjerum et al., 2020). Our study reports higher AUC values for all three groups using either pattern or ROI-based SVM classifiers (0.89 and 0.94 for AD; 0.90 and 0.93 for DLB; 0.98 and 0.99 for FTD, respectively).

Even though machine learning algorithms can achieve high diagnostic accuracy, their interpretation is not trivial. Understanding of the decision-making process behind the machine learning algorithm is essential for a wider acceptance among physicians (Burgos and Colliot, 2020) and different approaches can be used to look inside the black box of a machine learning model. In our study, we examined feature importance in three steps. First, we looked at the entire dataset and calculated feature importance based on a distance metric algorithm, and then repeated this procedure in the training set in each iteration. Based on feature weights and frequency histogram, we saw bilateral angular gyri, calcarine sulci, pons, cingulum, parts of frontal and occipital cortices, and inferior temporal and inferior parietal lobes as the most important features to make a prediction. However, this approach is unable to determine class-specific feature relevance, and thus we employed Shapley Additive exPlanations (SHAP; Lundberg and Lee, 2017). SHAP has gained popularity in machine-learning literature in recent years (Lundberg and Lee, 2017; Lundberg et al., 2020), but its usefulness in explaining machine learning models in neuroimaging field has not been fully utilized. By plotting the Shapley values, we could observe class-specific region importance, based on which the final prediction of the SVM model was made, as shown in Figure 8.

Visualization of class-specific regional importance showed characteristic and well-known changes in FTD (Brown et al., 2014) in bilateral frontal cortices. In DLB, we saw the importance of occipital changes and changes in medial temporal structures. The occipital hypometabolism is a known feature of DLB (McKeith et al., 2017). However, based on Shapley’s values, we cannot make a statement on the directionality of observed feature importance; the preservation of medial temporal structures is a consistent finding when comparing DLB to AD (Watson and Colloby, 2016). Conversely, in the AD group, our analysis did not reveal a classical pattern of hypometabolism in temporoparietal cortices and precuneus (Brown et al., 2014), presumably because similar regions are also affected in DLB (Perovnik et al., 2022b) and FTD (Nazem et al., 2018), and are thus lacking differential diagnostic value. Interestingly, the metabolic status of parietal lobes was deemed as the most important for classifying a scan as NC. The four patterns, shown in Figure 8, resemble classic metabolic brain patterns characterized with a more typical multivariate approach, such as PCA. However, multi-class SVM aims to separate the four groups, unlike the typical PCA analysis, which maximize the difference between diseased and healthy individuals. Highlighted class-specific regional importance can provide useful information also to an expert reader, who could in uncertain situations focus his or her attention to the regions bearing the most differential diagnostic information, instead of looking for a fully characterized pattern of neurodegeneration. Furthermore, an expert reader might benefit from using the output from the automated algorithm in conjunction with visual assessment. We hypothesize that this approach would provide the highest diagnostic accuracy. However, this remains to be addressed in future studies.

This study is not without its limitation. The patients included did not have a pathologically confirmed diagnosis. The accuracy of the diagnosis was improved with long-term follow-up by a dementia specialist in DLB and FTD groups and confirmed with CSF biomarkers in the AD group, which are in close concordance with pathological findings (Toledo et al., 2012). Clinicians making the diagnosis were not blinded to the visual reading of the FDG PET scan. While this could potentially be a source of bias, we included only those patients who fulfilled clinical diagnostic criteria in combination with other objective markers, i.e., CSF biomarkers in AD and dopamine transporter imaging in DLB. Furthermore, all subjects were scanned with the same PET scanner and the generalization of our findings to a more heterogeneous group remains to be tested. Some of the studied groups in our sample were small and we had class imbalance in our dataset. The latter was addressed by comparing the models based on the F1 scores, which are a more robust metric than accuracy in imbalanced datasets. The groups differed in their mean age and sex distribution with FTD and NC participants being younger than the other groups and the DLB cohort included more male participants than the NC group. These differences could have introduced a subtle bias to our models. Thus, the extension of the dataset to include a larger sample will be needed to evaluate this possibility.

## Conclusion

This study shows that a multi-class SVM algorithm based either on the expression of characteristic metabolic brain patterns or ROI glucose uptake can perform better than experts’ visual reading in the differential diagnosis of most common dementia syndromes using FDG PET scans. Furthermore, we have shown the utility of Shapley’s values for showing class-specific regional importance in explaining SVM models. A head-to-head comparison of interpretable machine learning model with visual reading has the potential of bringing computer-aided diagnosis closer to clinical workup. A future comparison with other imaging modalities and other diagnostic tools would be interesting.

## Data availability statement

The original contributions presented in the study are included in the article, further inquiries can be directed to the corresponding author.

## Ethics statement

The studies involving human participants were reviewed and approved by Slovenian National Medical Ethics Committee (no. 0120-584/2019/5). The patients/participants provided their written informed consent to participate in this study.

## Author contributions

MP, DE, and MT conceptualized the study. MP and TR collected the data. MP performed analyses and wrote original draft. AV, NN, and DE supervised analyses and methodology. JJ supervised data collection imaging procedure. MT, JJ, TR, and MP performed visual assessments of the scans. MP, CT, AV, NN, DE, and MT contributed to interpretation of the results and manuscript preparation. All authors contributed to the article and approved the submitted version.

## Funding

This work was supported by the Slovenian Research Agency (ARRS) through grant P1-0389 and projects J7-2600 and J7-3150.

## Conflict of interest

The authors declare that the research was conducted in the absence of any commercial or financial relationships that could be construed as a potential conflict of interest.

## Publisher’s note

All claims expressed in this article are solely those of the authors and do not necessarily represent those of their affiliated organizations, or those of the publisher, the editors and the reviewers. Any product that may be evaluated in this article, or claim that may be made by its manufacturer, is not guaranteed or endorsed by the publisher.
